# Drug-Resistant Tuberculosis on the Balkan Peninsula: Determination of Drug Resistance Mechanisms with Xpert MTB/XDR and Whole-Genome Sequencing Analysis

**DOI:** 10.1128/spectrum.02761-22

**Published:** 2023-03-06

**Authors:** Sara Truden, Eva Sodja, Manca Žolnir-Dovč

**Affiliations:** a University Clinic of Respiratory and Allergic Diseases Golnik, Golnik, Slovenia; National Center for Biological Sciences

**Keywords:** Xpert MTB/XDR, XDR, *Mycobacterium tuberculosis*, Balkan, drug resistance, molecular diagnostics, whole-genome sequencing

## Abstract

The new molecular assay Xpert MTB/XDR (Cepheid, Sunnyvale, CA, USA) was launched in 2021 to detect Mycobacterium tuberculosis (MT) complex with mutations conferring resistance to isoniazid (INH), ethionamide (ETH), fluoroquinolone (FQ), and second-line injectable drugs (SLIDs). The aim of our study was to evaluate the performance of the Xpert MTB/XDR rapid molecular assay on rifampicin-resistant, multidrug-resistant, and pre-extensively resistant tuberculosis (TB) isolates in a clinical laboratory in the Balkan Peninsula compared to a phenotypic drug susceptibility test (pDST). Xpert MTB/XDR was used to test positive Bactec MGIT 960 (Becton, Dickinson and Co., Franklin Lakes, NJ, USA) cultures or DNA isolates. In the case of discrepant results between Xpert MTB/XDR and pDST, the usefulness of whole-genome sequencing (WGS) was emphasized. In our study, 80 MT isolates from different Balkan countries were selectively chosen from the National Mycobacterial Strain Collection in Golnik, Slovenia. Isolates were tested with the Xpert MTB/XDR assay, conventional pDST, and WGS. Xpert MTB/XDR showed high sensitivities of 91.9%, 100%, and 100% for detecting INH, FQ, and SLID resistance, respectively, compared to pDST. In contrast, low sensitivity (51.9%) for ETH resistance was achieved because isolates harbored widespread mutations across the *ethA* gene. The specificity of Xpert MTB/XDR was 100% for all drugs except for INH (66.7%). Further investigation with WGS revealed −57c→t mutations in the *oxyR-ahpC* region marked with uncertain significance, which caused the low specificity for detecting INH resistance with the new assay. Xpert MTB/XDR can be used in clinical laboratories for the rapid detection of INH, FQ, and SLID resistance. Moreover, it can be used to rule in resistance to ETH. Additional use of WGS is recommended in cases of discrepant results between pDST and Xpert MTB/XDR. Future improvements of Xpert MTB/XDR with the inclusion of additional genes may increase the usefulness of the assay.

**IMPORTANCE** The Xpert MTB/XDR was tested on drug-resistant Mycobacterium tuberculosis complex isolates from the Balkan Peninsula. Positive Bactec MGIT 960 cultures or DNA isolates were tested as starting material. According to the results of our study with Xpert MTB/XDR, sensitivities for the detection of SLID, FQ, and INH resistance were sufficient (>90%) for the assay to be implemented into diagnostic algorithms. In our study, WGS revealed lesser-known mutations in genes conferring INH and ETH resistance, and their impact on resistance is still unknown. Mutations in the *ethA* gene causing resistance to ETH were scattered along structural gene without high-confidence markers for resistance. Therefore, resistance to ETH should be reported based on a combination of methods. Because the Xpert MTB/XDR assay was found to have good performance, we propose that it should be the method of choice for confirming resistance to INH, FQ, and SLID and conditionally for resistance to ETH.

## INTRODUCTION

Tuberculosis (TB) caused by Mycobacterium tuberculosis remains a global health problem despite a large decrease in the number of people with newly diagnosed TB in 2020 (5.8 million compared to 7.1 million in 2019) due to the COVID-19 pandemic (1). Early and reliable diagnosis of resistant TB is key to enrolling patients into an effective treatment regimen. Because many low-income countries are not able to perform phenotypic drug susceptibility testing (pDST), and pDST results may take weeks to obtain, treatment choice is often empirically based on past medical history and local prevalence of resistance (2). Since 2010, the World Health Organization (WHO) has recommended the use of the Xpert MTB/RIF (Cepheid, Sunnyvale, CA, USA) assay in the near-point-of-care setting. There is also a large population, estimated to be approximately 1.1 million people, infected with isoniazid (INH)-resistant TB, which is mostly undetected (3). A study by Heyckendorf et al. (2) showed that treatment regimens based only on Xpert MTB/RIF results can lead to suboptimal therapy for patients with multidrug-resistant (MDR) or extensively drug-resistant TB (XDR) TB compared to regimens based on pDST results. Until now, additional pDST or line probe assays (LPAs) have been necessary to exclude resistance to INH, fluoroquinolones (FQs), or second-line injectable drugs (SLIDs, including amikacin [AMI], kanamycin [KAN], and capreomycin [CAP]), which is important in the treatment of drug-resistant (DR) TB (4).

Therefore, the Xpert MTB/XDR (Cepheid) cartridge-based test was developed, which is suitable for near-patient testing because there are no requirements for specialized infrastructure. Tests can be performed directly from unprocessed sputum, processed sputum, or positive Bactec MGIT 960 (MGIT) (Becton, Dickinson and Co., Franklin Lakes, NJ, USA) cultures, and resistance to INH, ethionamide (ETH), FQ, and SLID can be determined by the presence of the M. tuberculosis complex (MTBc) in the sample (5, 6). This assay is intended to be used as a reflex test for a specimen that is determined to be M. tuberculosis-positive. The test can detect low or high levels of resistance to INH based on specific mutations in the *inhA* promoter region and mutations in *fabG1*, the *oxyR-ahpC* intergenic region, or *katG*. Moreover, mutation in the *inhA* promoter region confers resistance to ETH. Different types of mutations in quinolone resistance-determining regions reflect low or elevated levels of FQ resistance. Tests can also identify cross versus individual resistance to SLIDs based on mutations in the *eis* promoter region and in the *rrs* gene (7).

On the other hand, the rapid development of whole-genome sequencing (WGS) has facilitated the interpretation of genetic patterns underlying phenotypic resistance. The WHO issued a standardized, comprehensive catalogue of mutations and their associations with drug resistance. This catalogue of high-confidence mutations will expand in the future, and the inclusion of isolates from different geographical regions will strengthen the power of WGS, as seen from our results. The role of many mutations remains unclear, and with further observations and studies, the interpretation of some mutations will be subject to change (8, 9). Currently, the use of WGS is limited because it requires a bacterial culture as a mandatory step for sequencing. Moreover, specialized knowledge and infrastructure are needed to perform WGS.

To date, there are a lack of data on the use of the Xpert MTB/XDR assay on resistant MT isolates from the Balkan Peninsula in comparison with pDST and WGS. In 2019, all former Yugoslavian countries, Slovenia, Bosnia and Herzegovina, Serbia, Montenegro, and North Macedonia, reported low TB incidence rates (4.9, 17.6, 13.4, 13.1, and 9.6, respectively). Countries reported sporadic cases of rifampicin-resistant (RR) TB and MDR TB. In 2019, only North Macedonia (*n* = 1) and Serbia (*n* = 2) reported MDR TB cases, and no XDR TB cases were reported in any of the listed countries (10). The aim of our study was to test the performance of the new Xpert MTB/XDR molecular assay and compare its results to those of conventional phenotypic testing in MGIT tubes as a reference standard. Because this study was designed as a retrospective study, the use of positive MGIT tubes and DNA isolates on the Xpert MTB/XDR assay was tested. The sensitivity and specificity of the assay were tested against conventional pDST. With Xpert MTB/XDR, we obtained insight into the local prevalence of genes causing resistance to INH. WGS was used to illustrate the genetic characteristics of isolates with discrepant phenotypic and Xpert MTB/XDR results. The advantages and disadvantages of Xpert MTB/XDR compared to pDST and WGS are discussed.

## RESULTS AND DISCUSSION

### Xpert MTB/XDR.

From 1995 to 2021, 48 Slovenian MT isolates and 32 isolates from other Balkan countries were systematically chosen for testing with the new Xpert MTB/XDR assay. The isolates included in our study represent complete capture of the genetic diversity of Slovenian MDR isolates (Fig. S1) (11). Data gathered from European Centre for Disease Prevention and Control (ECDC) annual reports from 1995 to 2021 showed that 44.4% and 40.0% of North Macedonian and Montenegrin MDR isolates, respectively, were tested (Fig. S1) (10). This also gives us good insight into the genetic pattern behind resistant isolates from these Balkan countries. Isolates from Bosnia and Serbia were not representative because these countries had many more reported cases in 1999 to 2015, so more isolates should be included to obtain a larger picture. Since both countries together reported more MDR cases than Slovenia, North Macedonia, and Montenegro together during the study years, our study does not represent complete capture for the Balkan region. Moreover, more FQ- and SLID-resistant isolates should be included in the study, which might change the sensitivity and specificity of the Xpert MTB/XDR assay.

Based on the results of pDST, the resistance profiles of all isolates were determined. Phenotypic DST was used as a reference standard against which the sensitivity and specificity of Xpert MTB/XDR were calculated. All 80 isolates were successfully tested with Xpert MTB/XDR and yielded positive results for the presence of MTBc bacteria. Analysis showed discrepant results between pDST and Xpert MTB/XDR for at least one drug in 22 isolates. WGS was used to help interpret discrepant results between pDST and Xpert MTB/XDR. Isolates were sequenced with a median read depth of 73,39× (range of 26.40× to −112.98×). The results from WGS analysis are shown in Tables 1 and 2.

**TABLE 1:** WGS analysis of isolates (*n* = 11) with discrepant results of Xpert MTB/XDR and pDST for INH^*a*^

Sample	Xpert MTB/XDR	pDST	WGS			
			*katG*	*fabG1-inhA*	*oxyR-ahpC*	*mshA*
SLO-1301	R	S	G570A^*b*^		–57c>t^*c*^	
SLO-629	R	S	G570A^*b*^		–57c>t^*c*^	
SLO-617	R	S	G570A^*b*^		–57c>t^*c*^	
SLO-777	R	S	G570A^*b*^		–57c>t^*c*^	
SLO-874	R	S	G570A^*b*^		–57c>t^*c*^	
SLO-1809	R	S	G570A^*b*^		–57c>t^*c*^	
SLO-2496	S	R	361_ins_1_g>gc		–93_ins_2_g>ggt^*c*^	
MK-1856	S	R	D142G^*c*^			
MK-2617	S	R	W90S^*b*^			
BIH-3199	S	R			248_del_1_tg>t^*c*^	
CG-3029	S	R	–7c>t			575065-576031-del-966nt^*d*^

a WGS, whole-genome sequencing; INH, isoniazid; pDST, phenotypic drug susceptibility testing; R, resistant; S, susceptible. Mutations interpreted according to 2021 WHO mutation catalogue.

b Another variant reported at same codon with uncertain significance.

c Mutation has uncertain significance.

d Mutation is not present.

**TABLE 2:** WGS analysis of isolates (*n* = 13) with discrepant results of Xpert MTB/XDR and phenotypic drug susceptibility testing for ethionamide^*a*^

Sample ID	Xpert MTB/XDR	pDST	WGS				
	Promoter *inhA*	ETH	*ethA*	*ethR*	*Ndh*	*mshA*	*fabG1-*
MK-3224	NEG	R	A381D^*b*^, G43A^*c*^				
MK-3823	NEG	R	C137W^*b*^				
BIH-3202	NEG	R	A19E^*b*^				
CG-2984	NEG	R			971_ins_2_g_gcc^*c*^		
CG-3029	NEG	R				575,065-576,031_del_966^*d*^	
SLO-828	NEG	R	K472STOP^*d*^				
SLO-2496	NEG	R					
SLO-3128	NEG	R	K472STOP^*d*^				
SLO-2749	NEG	R	K472STOP^*d*^				
SLO-625	NEG	R	K472STOP^*d*^				
SLO-423	NEG	R	K472STOP^*d*^				
SLO-574	NEG	R	K472STOP^*d*^				
SLO-4711	NEG	R					c-34>t^*c*^, G241G^*c*^

a WGS, whole-genome sequencing; ETH, ethionamide; pDST, phenotypic drug susceptibility testing; R, resistant. Mutations interpreted according to 2021 WHO mutation catalogue.

b Another variant reported at same codon with uncertain significance.

c Mutation has uncertain significance.

d Mutation is not present.

One of the first prospective studies from Penn-Nicholson et al. on the use of Xpert MTB/XDR demonstrated high diagnostic accuracy for the detection of INH, FQ, and SLID resistance. Sensitivity and specificity did not differ between sputum samples and culture isolates (12). In our study, only a limited number of clinical specimens were included. Most of our specimens were either MGIT subcultures or DNA isolates; therefore, the sensitivity and specificity of the method cannot be compared regarding the type of specimen (Table S2). Penn-Nicholson et al. reported high specificity (>98%) for all drugs included in Xpert MTB/XDR, but sensitivity varied from 54% for ETH to 94% for FQ (12). The same study did not report false-positive results for detecting INH resistance and highlighted that this assay meets the minimum criteria set by the WHO for next-generation DST for FQ and INH. In contrast, the results from our study showed high specificity for all drugs (100%), except for INH, for which the specificity achieved was only 66.7% (Table 3). One possible explanation for the low specificity for detecting INH resistance is that 6/18 isolates were phenotypically sensitive to INH, where mutations in the *oxyR-ahpC* region were detected with Xpert MTB/XDR (Table 1). Repeated pDST for INH showed no resistance to INH. WGS was performed on the previously mentioned isolates. The results showed all isolates harboring the same mutation in the promoter region of the *oxyR-ahpC* –57c>t gene, which is listed as having uncertain significance in the 2021 WHO mutation catalogue (Table 1) (9). Moreover, the use of PhyResSE version 1.0 software also reported the isolates as being resistant. Epidemiological data together with genotyping showed that all 6 isolates were epidemiologically connected and shared the same genotype. Samples were isolated from patients in the same geographic region of Slovenia. Prior to this study, these isolates were reported to clinicians as being phenotypically monoresistant to pyrazinamide (PZA). Among the 6 false positives with Xpert MTB/XDR and WGS isolates, no relapse cases were reported. Two molecular tests recognized the isolates as resistant, but because pDST is the reference standard used in our study, this indicates that further investigation of mutations in the *oxyR-ahpC* region in correlation with INH resistance is needed.

**TABLE 3:** Xpert MTB/XDR results compared to conventional phenotypic drug susceptibility testing in Bactec MGIT 960 tubes^*a*^

Drug	Xpert MTB/XDR gene(s) covered	pDST				Total	Sensitivity (%)	Specificity (%)	Notes
		Sensitive isolates (*n*)		Resistant isolates (*n*)					
		Mutation not detected	Mutation detected	Mutation not detected	Mutation detected				
INH	promoter *inhA*, *katG*, *oxyR-ahpC*, *fabG1*	12	6	5	57	80	91.9	66.7	-
ETH	promoter *inhA*	46	0	13	14	73	51.9	100	7 isolates do not have pDST for ETH
FQ	*gyrA*, *gyrB*	65	0	0	7	72	100	100	8 isolates do not have pDST for FQ
SLID	*rrs*, *eis*	65	0	0	5	70	100	100	10 isolates do not have pDST for SLID

a INH, isoniazid; ETH, ethionamide; FQ, fluoroquinolones; SLID, second-line injectable drug; pDST, phenotypic drug susceptibility test.

In our study, Xpert MTB/XDR showed lower (91.9%) sensitivity for detecting INH resistance compared to a study from Penn-Nicholson et al. but was still sufficient to meet minimum criteria (>90%) for a next-generation DST set by the WHO (Table 3) (12). Five false-negative isolates out of 62 with confirmed phenotypic resistance to INH were subjected to WGS. The WGS results are presented in Table 1. In 1/62 isolates, a frameshift mutation in *katG* together with an insertion in the *oxyR-ahpC* region was detected. The latter is listed as having uncertain significance in the 2021 WHO mutation catalogue, but frameshift mutations in *katG* are high-confidence markers for INH resistance (8, 9). Moreover, 1/62 isolates had a mutation in the promoter region of *katG* (–7 C→T) in combination with a 966-nucleotide deletion in the *mshA* region, which causes a frameshift mutation. A study from Jagielski et al. (13) reported one MDR-TB isolate with a frameshift mutation in the *mshA* gene having MIC for INH of >5 mg/L with the proportion method on LJ. Moreover, the *katG* promoter region is a genomic region of interest for drug resistance in M. tuberculosis (14). Two INH-resistant isolates had a single mutation in *katG* (D142G, W90S) and one in the *oxyR-ahpC* region (248_del_1_ TG>T), all listed as having uncertain significance in the 2021 WHO mutation catalogue (9). In conclusion, five false-negative isolates with Xpert MTB/XDR harbored uncommon mutations that are not graded as high-confidence markers for resistance and are outside the coverage of Xpert MTB/XDR. Isolates with these mutations would be missed by other targeted molecular assays, such as line probe assays or targeted sequencing.

Resistance to INH is caused by mutations in various genes, and Xpert MTB/XDR covers the most frequently encountered regions (*katG*, *inhA*, *oxyR-ahpC*, *fabG1)*. The proportion of isolates with specific mutations varies depending on geographic location, with Africa having the highest percentage of *katG315* mutants and Asia having the lowest. On the other hand, Europe has the highest percentage of *inhA-15* mutants, 37.5% (15). Because our study is the first in the geographic region of the Balkan Peninsula, the distribution of mutations conferring INH resistance was estimated in our study. The results are presented in Table 4. According to the Xpert MTB/XDR results, mutations in the *katG* gene most frequently caused resistance to INH among our isolates (38/57; 66.7%), followed by mutations in the promoter regions of *inhA* (14/57; 24.6%) and *oxyR-ahpC* (7/57; 12.3%), with two isolates having double mutations in *katG* and the *inhA* promoter region. None of the isolates harbored mutations in *fabG1.* In comparison to other studies, where *katG* mutations have been detected in more than 85% of INH-resistant isolates, the frequency of *katG* mutants among our isolates was much lower (15, 16). A systematic review of mutations associated with INH resistance by Valafar in 2021 (15) showed a prevalence of mutations in *katG* loci at codon 315, with the variant S315T present in 66.23% of isolates. Locus 315 has the highest frequency of mutations globally, followed by the −15 and −8 promoter regions of *inhA*. In our study, a high frequency of mutations in the *oxyR-ahpC* region was detected, but a comparison cannot be made since no data are available regarding the prevalence of mutations in the *oxyR-ahpC* region for Europe (15).

**TABLE 4:** Distribution of mutations in genes conferring isoniazid resistance in 57 isoniazid-resistant isolates^*a*^

No. (%) of INH-resistant isolates detected with Xpert MTB/XDR				
Mutation detected in gene target				
Promoter *inhA*	*katG*	*oxyR-ahpC*	*fabG1*	Total
14^*a*^ (24.6)	38^*a*^ (66.7)	7 (12.3)	0 (0)	57^*a*^ (100)

a Two isolates had a double mutation in promoter *inhA* and *katG*.

Recommended methods for reliable detection of ETH resistance include phenotypic and genotypic testing. Penn-Nicholson et al. reported low sensitivity (54%) for the detection of ETH resistance using Xpert MTB/XDR (12). The low sensitivity for ETH observed in studies is due to the test limitations of the Xpert MTB/XDR assay, which only covers mutations in the promoter region of *inhA.* Other mechanisms and genes (*ethA*, *ethR*, *ndh*, *mshA*) are known to confer ETH resistance (17). In a systematic review, Enkirch et al. (18) reported low sensitivity (57.1%) for predicting ETH resistance with WGS. Both studies emphasize insufficient knowledge about the correlation between phenotype and genotype, as no graded list of *ethA* mutations is available (12, 18). Similar findings were observed in our study. The sensitivity for detecting ETH resistance was 51.9%, and the specificity was 100% (Table 3). The frequency of mutations in the *inhA* promoter region among ETH-resistant isolates was 51.8%, which is in proportion with other studies (17, 19). Therefore, 13/27 (48.1%) isolates had mutations causing ETH resistance outside the promoter region of the *inhA* gene (Table 2). The results of WGS showed no high-confidence mutations conferring ETH resistance; the mutations detected were reported in the WHO mutations catalogue as having uncertain significance or were not reported in the catalogue at all (Table 2) (9).

The proportion of ETH-resistant isolates harboring mutations in the *ethA* gene in our study was 9/27 (33.3%), and mutations were widespread across the structural gene (Table 2). Our findings are concordant with other studies in which mutations in *ethA* causing ETH resistance were widespread across structural genes and were found in 37% to 72% of isolates (17, 19–21). The results of WGS performed in 6/13 resistant isolates not detected with Xpert MTB/XDR showed a mutation in the *ethA* gene at codon 472, resulting in a stop codon (Table 2). According to a study by Johnsen et al. (14), the role of premature stop codons in *ethA* is not clearly understood, as it is in the case of *katG* or *pncA*. Stop codons were found in either ETH-susceptible or ETH-sensitive isolates (14). Since no other mechanisms of resistance were recognized in these 6 isolates, we suggest that the stop codon is the cause of resistance. These isolates are also epidemiologically connected and were involved in a microepidemic of MDR-TB in Slovenia (data not shown). In 4/13 isolates resistant to ETH, no mutation in *ethA* or in the *inhA* promoter region was found. Therefore, we searched other genes (*ndh*, *mshA*, and the *fabG1-inhA* intergenic region) and detected an insertion in *ndh* (1/27), a single-nucleotide polymorphism (SNP) in *fabG1* (1/27) and a deletion in *mshA* (1/27), but these are listed as having uncertain significance in the 2021 WHO mutation catalogue or are not in the catalogue at all (Table 2) (9). According to Vilcheze et al. (22), mutations in the *mshA* gene can cause resistance to ETH. Ultimately, only one isolate resistant to ETH had no known mutation in the examined regions.

In summary, even if the *ethA* gene was included in the kit, our isolates harbored uncommon mutations that would still have been missed. Xpert MTB/XDR has a low sensitivity (51.9%) for detecting ETH resistance and can be used to rule in ETH resistance, but it cannot rule it out (12, 18). Since ETH is suggested as an alternative to linezolid at 9 months, all-oral bedaquiline-containing treatment regimens and mechanisms of resistance are not well understood, and more studies should be carried out (23). Additionally, no criteria for sensitivity or specificity have been defined to date for a next-generation DST assay detecting ETH resistance (12).

Xpert MTB/XDR meets the minimum WHO criteria for a next-generation DST assay to be used at peripheral microscopy centers for the detection of FQ resistance (>95% sensitivity) (12). The sensitivity and specificity for detecting FQ resistance was 100% in our study. All 7 samples phenotypically resistant to FQ were confirmed with Xpert MTB/XDR (Table 3). One isolate harbored a low level of FQ resistance, and none had mutations in the *gyrB* gene. Even in countries where 25 to 31% of newly diagnosed TB isolates are FQ resistant, mutations in *gyrB* are rare (3.1%, 1.1%) (24, 25). Among our isolates, three had *gyrA1* and *gyrA3* mutations, two had *gyrA1*, *gyrA2*, and *gyrA3* mutations, one had *gyrA1* and *gyrA2* mutations, and one had only a mutation in the *gyrA3* region. Isolates with *gyrA1* and *gyrA2* mutations had low-level resistance to FQ, and WGS detected the mutation D94A in the *gyrA* region (data not shown). With a new all-oral 6-month treatment regimen, access to rapid DST for ruling out FQ resistance is emphasized (23). On the other hand, SLIDs are excluded from the new all-oral 6-month treatment regimen; however, including them in the Xpert MTB/XDR rapid molecular test is still valuable when countries switch to a new regime or if the regimen cannot be assembled (12). A low number (*n* = 5) of isolates resistant to SLID were included in our study, with two having mutations in the *eis* gene and three having mutations in the *rrs* gene. The sensitivity and specificity were 100% (Table 3). In the study by Penn-Nicholson et al., the sensitivity for the detection of resistance to CAP, AMI, and KAN varied from 60 to 86%, while the specificity was 100% for AMI and CAP resistance and 98% for KAN resistance (12). Phenotypic resistance to AMI, KAN, and CAP is associated with a larger diversity of genes. According to a systematic review from Georghiou et al. (26), a mutation in *rrs* 1401 alone can predict 70 to 80% of AMI- and CAP-resistant strains and 60% of KAN resistance cases. Our strains with mutations in the *eis* gene were both resistant to KAN, and one had additional resistance to AMI. Strains with *rrs* mutations were resistant to either CAP or AMI. More isolates resistant to SLIDs should be included to better capture the resistance patterns of SLID-related genes in the Balkan Peninsula.

To conclude, the major advantage of Xpert MTB/XDR is its use in the near point of care setting without the need for bacterial culture. Since the pDST procedure is more complex and may take weeks to obtain results, the use of Xpert enables the early diagnosis of INH-resistant TB and thus the rapid allocation of patients to the appropriate treatment regimen. On the other hand, WGS enables insight into the whole genome of bacteria, and other mechanisms of resistance can be detected that are not included in the Xpert panel. One of the benefits of this study is finding SNPs with lesser-known impact on resistance to ETH and INH and emphasizing the importance of comparative studies at different geographical settings with the use of different diagnostic tools. The sensitivity for detecting ETH resistance was suboptimal due to assay limitations because only mutations in the *inhA* promoter region are included. Moreover, in the case of ETH resistance, we propose the use of a combination of molecular methods (Xpert MTB/XDR and WGS) and conventional pDST. The main disadvantage of the Xpert assay is the inability to detect mutations for new or repurposed drugs (bedaquiline, delamanid, pretomanid, clofazimine, linezolid) that are included in the treatment of drug-resistant TB (6). Improvements of the Xpert assay should include INH and rifampicin (RIF) resistance-related genes in one cartridge, which will facilitate the diagnosis of MDR TB in the clinical laboratory.

## MATERIALS AND METHODS

### Strain collection.

Isolates included in our study were retrospectively and selectively chosen and retrieved from the Slovenian National Mycobacterial Strain Collection. Overall, 80 isolates were included, 48 of which were collected from clinical specimens of Slovenian patients. The isolates were processed as part of routine diagnostic procedures between 1995 and 2021 in the Laboratory for Mycobacteria (University Clinic Golnik, Golnik, Slovenia). The remaining 32 isolates were sent to our laboratory from different Balkan countries to assist with pDST from 1999 to 2015. These were isolated from North Macedonian (*n* = 24), Montenegrin (*n* = 4), Bosnian (*n* = 3), and Serbian patients (*n* = 1). Isolates were identified as either sensitive or mono-, poly-, or MDR-resistant based on phenotypic DST. Among the 48 Slovenian isolates, there were 22 MDR cases, which represent 75.9% of all MDR isolates from 1995 to 2021 (Fig. S1) (11). The percentages of isolates tested from other Balkan countries represent 44.4% of North Macedonian, 40.0% of Montenegrin, 3.1% of Bosnian, and 0.79% of Serbian MDR or XDR isolates from 1999 to 2015 (10).

### Phenotypic testing.

All M. tuberculosis isolates (*n* = 80) were tested for drug susceptibility to first-line drugs (INH 0.1 μg/mL, RIF 1.0 μg/mL, ethambutol 5.0 μg/mL, PZA 100.0 μg/mL, streptomycin 1.0 μg/mL); 73 were tested for ETH (5.0 μg/mL), 70 for SLID (AMI, KAN, CAP), and 72 for FQ resistance (ciprofloxacin [CIP], ofloxacin [OFL], moxifloxacin [MXF], and levofloxacin [LFX]). Phenotypic drug susceptibility testing was performed using the Bactec MGIT 960 System (Becton, Dickinson, and Co., Franklin Lakes, NJ, USA) according to the manufacturer’s procedures (27). All 80 isolates included in the study underwent pDST for INH, and 62/80 showed resistance to INH. Out of 80 isolates, 73 had pDST performed for ETH, and 27 were phenotypically resistant to ETH. Phenotypic DST for FQ (CIP, OFL, MXF, LFX) was performed on 72/80 isolates, 7 of which showed resistance to FQ. Phenotypic testing for SLID was performed on 70 isolates, 5 of which showed resistance to either AMI, KAN, or CAP. A total of 9/80 (11.3%) isolates were found by pDST to be sensitive to first-line drugs, FQs, and SLIDs (Table S2).

### DNA isolation.

DNA was isolated from colonies grown on Löwenstein-Jensen slopes. Two full loops of bacterial culture were collected into 1× Tris-EDTA (TE) buffer and heat-inactivated for 20 min at 80°C. Afterwards, the samples were processed using the cetyltrimethylammonium bromide method according to previously described procedures (28). DNA isolates were stored in 1× TE buffer at −20°C until they were used for further analyses. Isolated DNA was quantified using Qubit 1× dsDNA HS Assay Kits with a Qubit 4 Fluorometer (Thermo Fisher Scientific, Waltham, MA, USA) (29), and purity was assessed spectrophotometrically using a NanoDrop 2000 (Thermo Fisher Scientific).

### Xpert MTB/XDR.

Xpert MTB/XDR is an automated *in vitro* diagnostic test based on nested real-time PCR for the detection of XDR MTBc DNA. It can be performed on unprocessed sputum samples, concentrated sediments prepared from sputum, or MGIT culture (5). In our study, the starting material for Xpert MTB/XDR was decontaminated sputum sediment in 3/80 samples, MTB-positive cultures from MGIT tubes in 36/80 samples, and DNA isolates in 41/80 samples. For the latter, 5 μL of DNA isolate was first added to 500 μL of phosphate-buffered saline (PBS) and then processed as decontaminated sputum samples according to the manufacturer’s procedures. Sputum samples and MGIT-positive cultures were processed according to the manufacturer’s procedures (5). The loaded cartridge was inserted into the GeneXpert instrument and, together with its software, the results were interpreted depending on the measured fluorescence signal and melting temperature values. All samples generated valid and positive MTBc results.

### Whole-genome sequencing.

DNA libraries were prepared with an Illumina DNA Prep kit (30, 31) and sequenced on an Illumina MiSeq platform with a MiSeq Reagent Micro kit v2 according to the manufacturer’s instructions (Illumina, San Diego, CA, USA). Library size was determined using an Agilent High Sensitivity DNA kit (Agilent Technologies, Santa Clara, CA, USA) according to the manufacturer’s procedures (32). The following promoter and/or coding regions were considered for ETH: Rv1483 promoter *inhA*, Rv3854*c ethA*, Rv3855 *ethR*, Rv0486 *mshA*, and Rv1854c *ndh*. For INH, the following regions were included: promoter *inhA*, Rv1483 *fabG1*, Rv1908c *katG*, Rv2427-Rv2428 *oxyR-ahpC* intergenic region, Rv1854c *ndh*, and Rv0486 *mshA*.

### WGS data analysis.

Twenty-two M. tuberculosis isolates were sequenced, and FASTQ files were analyzed using the PhyResSE v1.0 online tool and MTBseq pipeline v1.0.4 (33). Sequencing reads were mapped against the reference genome H37Rv (GenBank accession no. NC_000962.3). The analysis was performed on the mapped MTBc reads with a quality threshold of a mean coverage of at least 30×. Parameters for the MTBseq pipeline called a variant only if a mutation was detected in at least 4 forward and 4 reverse reads with a Phred quality score of 20 and mutation frequency of ≥75%.

### Statistical analysis.

The sensitivity and specificity of Xpert MTB/XDR were calculated for INH, ETH, FQ, and SLID resistance based on the results of conventional pDST in MGIT, which was used as a reference standard. Sensitivity was defined as the proportion of resistant isolates detected with pDST and confirmed with Xpert MTB/XDR. Specificity was calculated as the proportion of phenotypically sensitive isolates that were identified as sensitive with Xpert MTB/XDR.

### Ethical approval.

The study was approved by the Slovenian National Medical Ethics Committee (approval no. 0120-94/2021/3).

### Data availability.

Sequences of 22 isolates were deposited in GenBank (https://www.ncbi.nlm.nih.gov/) under the accession no. PRJNA851574.
