# Supplementary material for: Drug-Resistant Tuberculosis on the Balkan Peninsula: Determination of Drug Resistance Mechanisms with Xpert MTB/XDR and Whole-Genome Sequencing Analysis
Source: Microbiol Spectr. 2023 Mar 6;11(2):e02761-22. doi: 10.1128/spectrum.02761-22 (PMC10100718; doi:10.1128/spectrum.02761-22)

Supplementary Figure S1: Total number of Slovenian MDR/XDR isolates and number of isolates included in our study in period from 1995-2021.

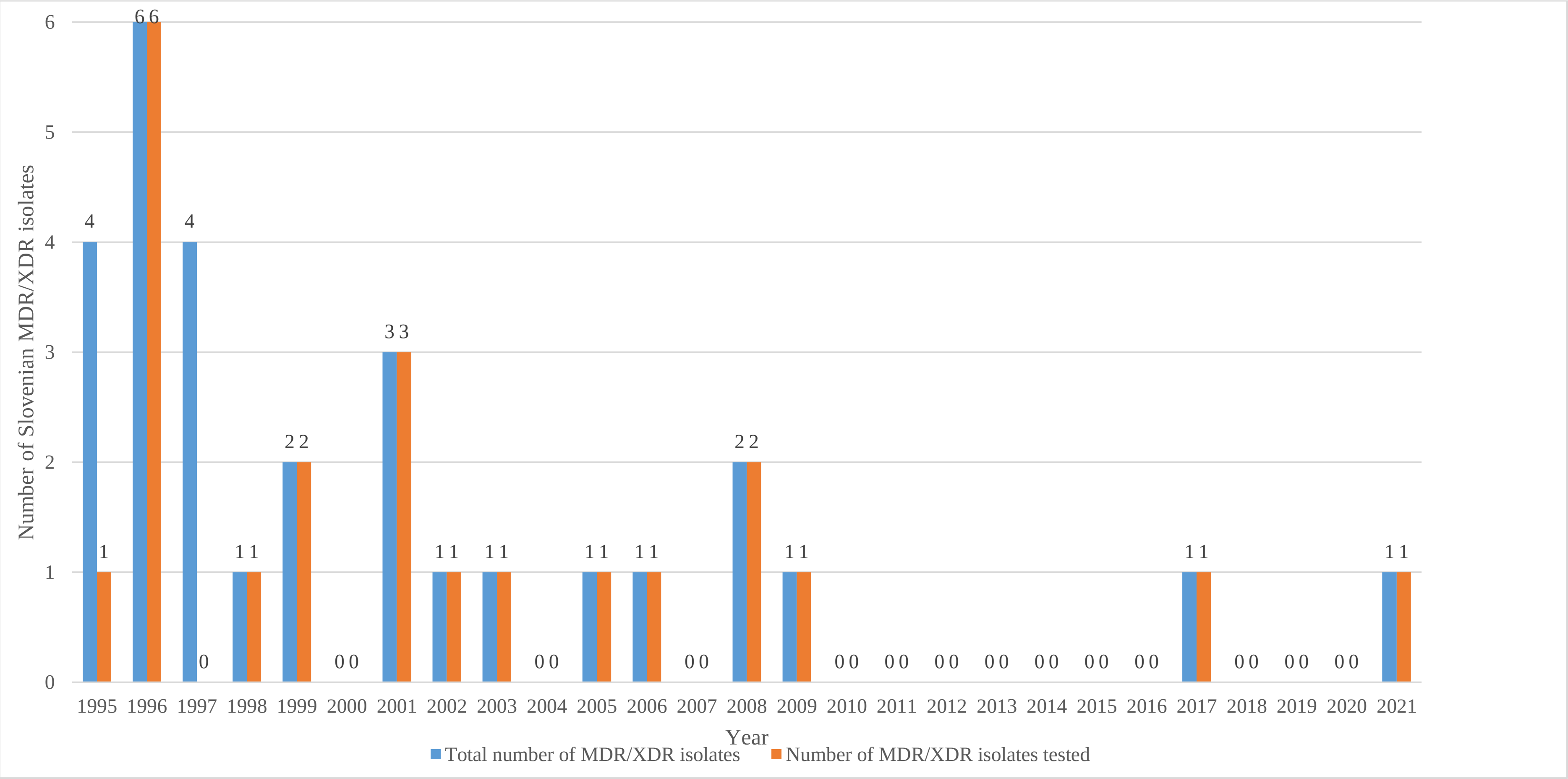

Supplement: Supplemental file 1 — Fig. S1. Download spectrum.02761-22-s0001.pdf, PDF file, 0.01 MB [file spectrum.02761-22-s0001.pdf]
